# Fine‐scale spatial genetic structure, mating, and gene dispersal patterns in *Parkia biglobosa* populations with different levels of habitat fragmentation

**DOI:** 10.1002/ajb2.1504

**Published:** 2020-07-07

**Authors:** Djingdia Lompo, Barbara Vinceti, Heino Konrad, Jérôme Duminil, Thomas Geburek

**Affiliations:** ^1^ Centre National de Semences Forestières 01 BP 2682 Ouagadougou 01 Burkina Faso; ^2^ Department of Forest Genetics Austrian Research and Training Centre for Forests Seckendorff‐Gudent‐Weg 8 1131 Vienna Austria; ^3^ Bioversity International Viale Tre Denari 472 00054 Maccarese Rome Italy; ^4^ UMR‐DIADE Institut de Recherche pour le Développement Univ. Montpellier Montpellier France; ^5^ Service Evolution Biologique et Ecologie, CP160 ⁄ 12 Faculté des Sciences Université Libre de Bruxelles 50 Av. F. Roosevelt 1050 Brussels Belgium

**Keywords:** gene conservation, non‐timber forest products, *Parkia biglobosa*, paternity analysis, reproductive biology, spatial genetic structure

## Abstract

**Premise:**

A good understanding of genetic variation and gene dispersal in tree populations is crucial for their sustainable management, particularly in a context of rapid environmental changes. West African Sudanian savannahs are being fragmented and degraded, partly due to expansion of crop cultivation and monocultures that reduce tree density and may impact pollinators. The population dynamics of important indigenous trees could also be affected. We investigated the influence of habitat fragmentation on patterns of genetic diversity and gene dispersal of a key Sudanian agroforestry tree species, *Parkia biglobosa*.

**Methods:**

Using 10 highly polymorphic nuclear microsatellites, we genotyped 2475 samples from reproductive trees, seedlings, and embryos in four tree populations presenting different levels of habitat fragmentation.

**Results:**

*Parkia biglobosa* presented similar high genetic diversity across the four populations studied. Genetic diversity and inbreeding were similar between adults and embryo cohorts. In all four populations, the selfing rate was less than 1%. The effective number of pollen donors per tree was high (*N*_EP_~ 18–22), as was the pollen immigration rate (from 34 to 74%). Pollen dispersal was characterized by a fat‐tailed distribution with mean estimates exceeding 200 m. In three populations, stem diameter had a pronounced effect on male reproductive success. Here, the highest male reproductive success was observed in trees with a diameter at breast height between 60 and 75 cm.

**Conclusions:**

At the scale analyzed, fragmentation does not seem to pose limitations to gene flow in any of the sites investigated, regardless of the landscape configuration associated with the different tree stands. The study provides useful insights on the reproductive biology of an important tree species in the West African savannahs.

A good understanding of genetic variation within and among populations of a species is a necessary foundation for its sustainable management, including the conservation of its genetic resources, particularly in the face of climate changes and increasing threats associated with habitat loss and fragmentation. Genetic diversity is a source of valuable traits; the main reasons for conserving it are to ensure the future adaptability of tree populations; preserve particular traits of interest for production, income, or cultural reasons; and maintain other valuable ecosystem services supporting the resilience of forests and landscapes to future environmental changes (Dawson et al., 2011; Jamnadass et al., 2011; Alfaro et al., 2014). The possibility of tapping into sufficiently diverse planting material at species and intraspecific levels is also crucial to sustaining successful forest restoration initiatives (Thomas et al., 2015; Broadhurst et al., 2016). The spatial distribution of the neutral genetic diversity in a tree species is determined by gene flow patterns, which play an important role in maintaining genetic connectivity within and between populations (Hamilton, 1999; Dick et al., 2008).

Agricultural intensification often implies a reduction of population densities of the naturally occurring species of the landscape, which implies an increase in the distance among congeneric individuals. This results in a fragmentation of the species' habitat with potentially strong impacts on their reproduction dynamics and, in turn, on their genetic makeup (Eckert et al., 2010; Leimu et al., 2010; Aguilar et al., 2019). The influence of population fragmentation effects, caused by anthropogenic changes on the fine‐scale spatial genetic structure and on gene flow, has been described for some temperate (e.g., Kamm et al., 2009; Dubreuil et al., 2010) and tropical trees (e.g., Dick et al., 2008). In sub‐Saharan Africa, the influence of fragmentation and/or forest exploitation has been studied for a number of timber species (Born et al., 2008; Bizoux et al., 2009; Ndiade‐Bourobou et al., 2010; Debout et al., 2011; Duminil et al., 2016a, b; Monthe et al., 2017) and riverine forest tree species in West Africa (Ewedje et al., 2017). However, very limited knowledge is available for African savannah trees supplying valuable non‐timber forest products. With this study, we tried to fill this gap, focusing on *Parkia biglobosa*, an important West African tree species, naturally widespread across West Sudanian savannahs (Hall et al., 1997) and well represented in traditional agroforestry systems (parklands). One of the main drivers at landscape level is cotton farming, reaching an annual production of 650,000 tons in Burkina Faso in 2006 (Vitale et al., 2011). Since then, the production area has fluctuated between 400,000 and 700,000 ha, positioning the country among the largest cotton producers in Africa (Pertry et al., 2016). However, this commodity represents a major threat to agroforestry tree species in the region (Gaisberger et al., 2017), potentially affecting the genetic diversity, mating system, and gene flow of agroforestry trees through at least two different mechanisms: (1) a reduction of tree population densities and (2) a potential negative impact on pollinators, due to heavy use of pesticides.

Agriculture practices cause a fragmentation of tree populations and a reduction in tree density that may affect tree mating patterns. Several studies indicate that fragmentation of the forest cover tends to increase selfing in the remaining tree individuals and mating among relatives, with a negative effect on progeny vigor and on the number of pollen donors (e.g., Fuchs et al., 2003; Manoel et al., 2012; Aguilar et al., 2019). Cotton plants do not thrive well under tree cover; consequently, its cultivation leads to a removal of trees, especially of large individuals (Bayala et al., 2011). A reduction in population density of a species may lead to decreased pollen‐mediated gene flow (either zoophilous or anemophilous), that consequently limits the possibilities for crossing among intraspecific congeners (Karron et al., 1995). However, different studies have demonstrated that the selfing rate of a tree species does not necessarily increase with a reduction in population density (Carneiro et al., 2011; Duminil et al., 2016a), due to the mitigating effect of the longer distance covered by pollen through dispersal mechanisms (Hardy et al., 2006). Counter‐intuitively, evidence has been provided that, in some cases, pollen‐mediated gene flow may even increase as a consequence of a reduced population density (e.g. Duminil et al., 2016a).

In animal‐pollinated plants, the type of pollinators and their richness also affect plant mating and gene flow. Plant reproductive success is determined by the ratio between self‐pollen (selfing) and outcross‐pollen (outcrossing). Selfing is generally detrimental, particularly in tree species, and leads to inbreeding depression (Geburek, 1986; Duminil et al., 2009). Reproductive success is further determined by the distance travelled by pollen, a variable dependent on the foraging behavior of pollinators in animal‐pollinated plant species. The loss or reduction of pollinator abundance can thus result in reduced seed set or even in complete reproductive failure (Bond, 1994; Kearns and Inouye, 1997). Plants pollinated by a large range of pollinators are a priori less affected by a decline in pollinators, and their pollen can be dispersed over a wider range of distances (Barthelmess et al., 2006; Hasegawa et al., 2015).

Pesticides have been reported among the causes of observed declines in pollinators (Potts et al., 2010) although the effects of pesticides are not always clear. Scientific results on the effects of pesticides are geographically specific, related to the characteristics of the mix of pollinators found in a specific region (De Palma et al., 2016). Moreover, results are largely based on investigations covering few regions of the world (mainly North America and Western Europe) and over‐representing some types of pollinators not spread across all continents (e.g., bumblebees are intensively studied but not present in Africa). In Burkina Faso, 90% of the agricultural pesticides applied are used for cotton cultivation (Ouedraogo et al., 2011) and mainly nonselective (especially insecticides), impacting nontarget pollinator species and potentially affecting pollination services (McCauley, 2003; Ouattara, 2007; Winfree et al., 2009; Abaga et al., 2011).

Levels of genetic diversity and the mating system and patterns of gene flow can be investigated using molecular markers. Codominant markers, such as microsatellites, are particularly useful in this context (Ouborg et al., 1999). Both indirect and direct methods can be used to characterize historical and contemporary gene flow in populations. One indirect method is based on the fine‐scale spatial genetic structure (SGS) of adult individuals to obtain information on historical gene flow (Vekemans and Hardy, 2004; Hardy et al., 2006). This method relies on a set of mapped and genotyped adult individuals to estimate the strength and the distance of overall gene dispersal (pollen‐ and seed‐mediated gene flow are confounded) (Hardy and Vekemans, 1999). Another indirect method relies on the SGS of pollen clouds to obtain information on contemporary gene flow (Robledo‐Arnuncio et al., 2007). As with the previous method, the advantage is that potential pollen donors do not need to be mapped and genotyped in the studied population, but the distribution of pollen dispersal distances is estimated from the geographical coordinates and genotypes of a set of progeny arrays (mother and offspring). Direct methods instead rely on parentage analyses, performed through a genetic analysis of adults and progeny arrays. In contrast to indirect methods, this approach requires an exhaustive sampling of potential paternal trees and the use of highly polymorphic markers to enable an accurate estimate of contemporary gene flow patterns (Marshall et al., 1998; Burczyk and Chybicki, 2004; Chybicki, 2018). In this case, both the accuracy and the effort are a priori higher than when using indirect methods (Smouse and Sork, 2004).

The present study investigated the level of genetic diversity and patterns of gene flow in an agroforestry tree species growing in landscapes with a different configuration of land uses, with variable levels of fragmentation of the suitable habitat of the tree species, depending on the degree of clearing for agriculture and cotton farming. We expected to detect differences in genetic diversity and gene flow in *P. biglobosa* populations interspersed across different farmland settings. To test this hypothesis, we selected four populations, two from cotton farms that had high levels of habitat fragmentation (named “cotton populations”, CP hereafter) and two from traditional agroforestry parklands with limited levels of fragmentation (“non‐cotton populations”, NCP) and compared the two clusters for the following traits: (1) levels of genetic diversity and inbreeding; (2) mating system (i.e. outcrossing rate); (3) contemporary gene dispersals (as inferred from the SGS of pollen clouds or from parentage analyses). Due to an effect of cotton cultivation on population density and pollinator abundance in CP, we expected: (1) a signal of decrease in genetic diversity and of increase in inbreeding between the adult and the embryo cohorts in CP, but no signal in NCP; (2) lower outcrossing rates in CP than in NCP; (3) lower pollen‐mediated gene flow in CP than in NCP.

## Materials and methods

### Species description

*Parkia biglobosa* (Jacq.) G.Don, commonly named African locust bean or néré (French), is a fruit tree species belonging to the Fabaceae, precisely to the mimosoid clade in the Caesalpinioideae subfamily, based on the most recent classification (LPWG, 2017). *Parkia biglobosa* occurs mainly in African savannahs between 4°N to 15°N of latitude from Senegal to Uganda (Hall et al., 1997). In addition to its ecological importance, this species plays an important economic and social role for rural communities by providing food, medicine, fodder, fuel wood, and soil fertilization (Ouedraogo, 1995; Hall et al., 1997). It is also a melliferous tree important for apiculture and a source of tannins for artisanal use (Ouedraogo, 1995). The species is monoecious, each tree bearing hundreds to thousands hermaphroditic capitula, each with about 2200 fertile female flowers, 80 staminoid (male) flowers, and 260 infertile nectar flowers (Hopkins, 1983). Pollen is shed in polyads, i.e., packages of 16 or 32 pollen grains (Baker and Harris, 1957). Despite the numerous fertile flowers, only up to 25 pods form on an infructescence (Ouedraogo, 1995). Each pod contains up to 24 seeds (Ouedraogo, 1995) whose embryos are sired by the same father (Lassen et al., 2014). Young trees start flowering and fruiting at the age of 8 years, corresponding approximately to a DBH of 10 cm (Lompo, 1999), and reach their full sexual maturity at 30–50 years of age (DBH of 35 cm). Flowering lasts 4 months during the dry season, starting in January and showing in February/March about 80% floral synchronism within population (Sina, 2006). A previous genetic study, based on allozyme markers, suggests that *P. biglobosa* is predominantly outcrossing (Sina, 2006). A controlled‐pollination trial showed that self‐pollination is possible, particularly through geitonogamy (Ouedraogo, 1995). The species is essentially animal‐pollinated, with about 30 potential pollinating species visiting its flowers (Lompo et al., 2017); bats, honey bees, and stingless bees are the most effective pollinators (Ouedraogo, 1995; Lassen et al., 2017). In addition to humans, seed dispersers are mainly primates, such as chimpanzees, small mammals (rodents), and birds (Ouedraogo, 1995).

### Site sampling and data collection

**Figure 1:**
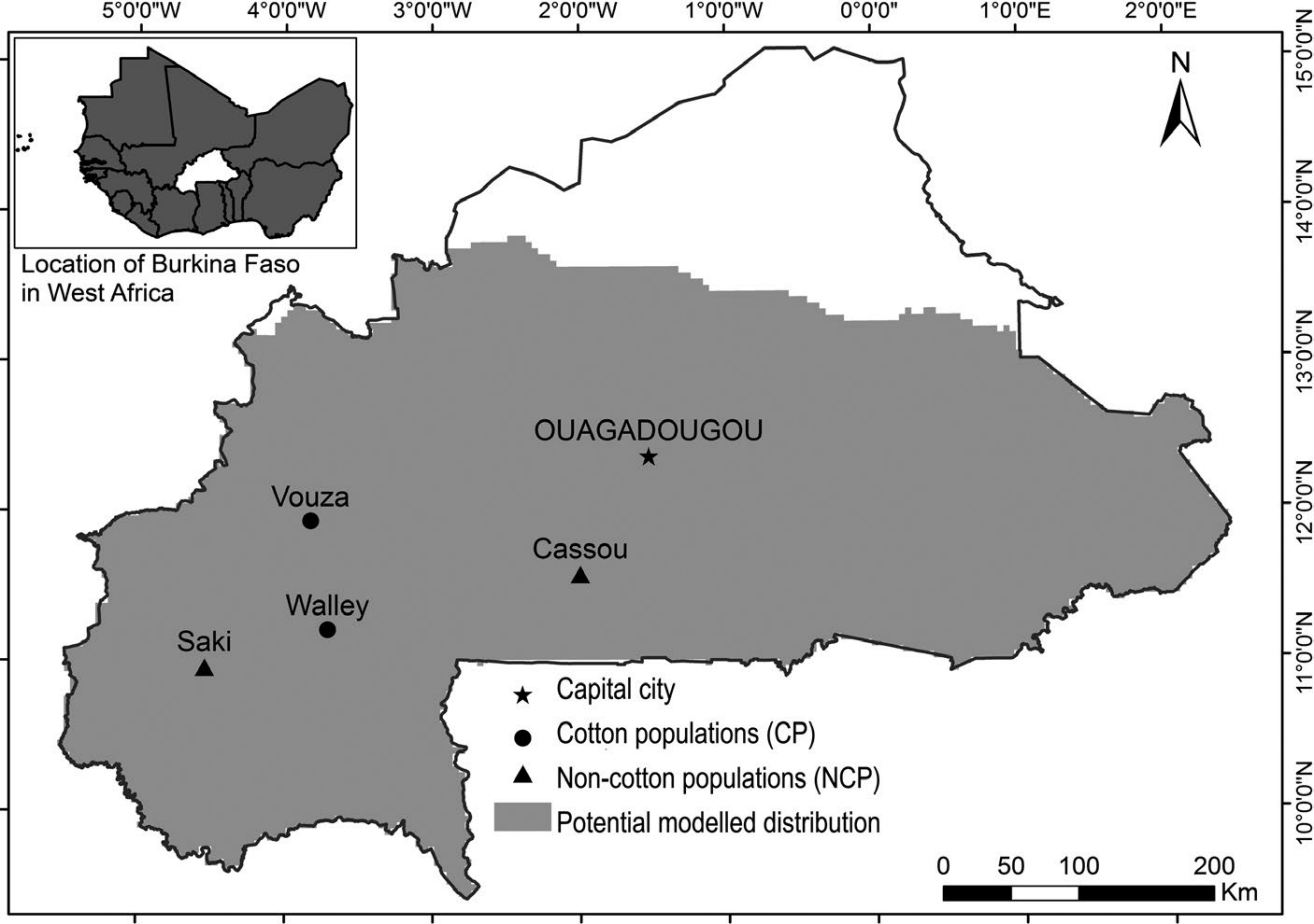
Location of 993 study populations within the modelled distribution map of *Parkia biglobosa* (modified from Gaisberger et al., 2017) in Burkina Faso (West Africa).

Our study was conducted in the south‐Sudanian region of Burkina Faso (Fig. 1), characterized by an annual rainfall from 900 to 1100 mm within a period of 5–6 months (Fontes and Guinko, 1995). We selected four continuous *P. biglobosa* populations that included a cultivated central area (hereafter, “plot”), where individuals of useful tree species—retained after clearing the land for agriculture—were interspersed among crops to form a so‐called parkland. The dominant tree species inside the plots was *P. biglobosa*, and the most‐represented associated tree species were medicinal and food trees such as *Adansonia digitata*, *Detarium microcarpum*, *Diospyros mespiliformis*, *Tamarindus indica*, *Vitellaria paradoxa*, and *Vitex doniana*. The four selected *P. biglobosa* populations differed in diameter structure, tree density, abundance of small and large trees, presence of regeneration (Table 1), and occurrence of cotton cultivation in the plot. All four populations were surrounded by an area with natural vegetation, consisting of a savannah with a mix of trees and shrubs. We selected two CP populations (Walley and Vouza), where cotton cultivation started in the early 1960s and the typical cultivation system is a cotton–maize rotation (the two crops are rotated on a yearly basis) with considerable input of pesticides for both crops.

**Table 1:** Characteristics of the four *Parkia biglobosa* populations investigated.

Population characteristics	Non‐cotton populations		Cotton populations	
	Saki	Cassou	Walley	Vouza
Latitude (°)	10.96	11.58	11.21	11.94
Longitude (°)	–4.54	–2	–3.71	–3.82
*N* large trees in plot (+)	42 (12)	45 (16)	83 (19)	106 (20)
*N* small trees in plot (+)	55 (6)	48 (4)	26	3
*N* seedlings in plot	7	18	0	0
*N* embryos	408	456	419	464
*N* adults in plot	97	93	109	109
Area of plot (ha)	6.7	11.2	23.4	60.8
Density (adults ha^‐1^)	14.5	8.3	4.7	1.8
Distance in m (mean; min; max)	(130; 2; 312)	(191; 1; 540)	(246; 2; 692)	(476; 6; 1203)
*N* large trees in transect	102	43	54	61
*N* small trees in transect	13	6	11	5
*N* adult trees in plot and transect	212	142	174	175

Notes

*N*, sample size; +, number of maternal trees given in bracket; Dist. in m, pairwise distance between individuals in meters for each plot (mean, minimum [min.] and maximum [max.] distance). Seedlings DBH < 10 cm, small trees 10 cm > DBH < 35 cm, large trees DBH > 35 cm.

The two NCP populations (Saki and Cassou) are located in areas where parklands were established in more recent times (1990s) and tree populations have never been mixed with cotton. Agriculture in these locations is a traditional farming system that is not based on pesticides use; annual crops (e.g., sorghum, maize, and millet) are cultivated in combination with a tree cover formed by useful tree species. We considered Cassou as a NCP, given that it is located 5 km from the first cotton field (the recent cotton company named Fasocotton was established in the area in 2004) and has a diameter structure that supports this classification (Appendix S1). In each population, we adapted the size of the plot in order to collect at least 100 adult individuals. Given the variation in population density, the size of the plot differed significantly among populations: 6.7, 11.2, 23.4, and 60.8 ha in Saki, Cassou, Walley, and Vouza, respectively (Table 1; Appendix S2). On the basis of pairwise distances between individuals in each plot, the population with the lowest density presented a mean distance (Vouza, 476 m) that was two, three, and four times higher compared with the other populations, respectively, Walley, Cassou, and Saki (Table 1). In each plot, we sampled and geo‐referenced all seedlings (DBH < 10 cm) and adult trees (small trees, 10 > DBH < 35 cm and large trees, DBH > 35 cm). The adult tree density in plot varied from 1.8 to 14.5 individuals per hectare (Table 1). In addition to sampled trees in the plot, other adult trees were sampled along approximately 5 km long paths departing from the plot in four directions (north, south, east, and west) (Appendix S2). Adult trees encountered along the paths were systematically sampled. Leaf samples from seedlings and adult trees were dried in silica gel and stored at 4°C until DNA extraction. Furthermore, we collected mature pods from 12 to 20 selected maternal trees in each plot during the optimal period of fruit maturation. In total, 18–24 pods were collected for each maternal tree, throughout the crown, by retaining only one separated pod per infructescence. Considering that all embryos in a pod are full siblings (Lassen et al., 2014), we sampled one seed per pod, then extracted and genotyped its embryo to achieve an unbiased representation of the diversity of contributing pollen sources. Genomic DNA was extracted from 3 to 4 leaflets per individual tree.

### DNA extraction and genotyping

Overall, DNA was isolated from seed and leaflet samples of 2475 individuals composed of 703 adult trees (167 small and 536 large trees), 1747 embryos, and 25 seedlings from Cassou and Saki. The total genomic DNA was extracted using the DNeasy 96 Plant Kit protocol (QIAGEN, Hilden, Germany) and the manufacturer's instructions. DNA samples were genotyped using 10 nuclear microsatellite loci (nSSR) developed by Lassen et al. (2014) and the method applied by Lompo et al. (2018).

### Genetic diversity and inbreeding analyses

We tested whether CP present a decrease in genetic diversity and an increase in inbreeding from the adult to the seed cohort stages (whereas no effect was expected for NCP). To this purpose, we estimated the following genetic diversity variables per population and per cohort in each population using the program SPAGeDi 1.4 (Hardy and Vekemans, 2002): number of observed alleles (NA), effective number of alleles (NA_E_) following Nielsen et al. (2003), rarefied allelic richness (*A*_R_) expressed as the expected number of alleles based on a minimum subsample size of *k* gene copies, expected heterozygosity (often called gene diversity) corrected for sample size (*H*_E_) (Nei, 1978), observed heterozygosity (*H*_O_), and individual inbreeding coefficient (*F*). We tested the significance of inbreeding coefficients using 10000 gene copy permutations. The presence of null alleles causes a bias in the proportion of heterozygosity and consequently affects the estimates of inbreeding coefficients within a population (Chybicki and Burczyk, 2009). Therefore, we calculated the null allele frequencies per locus and the inbreeding coefficients accounting for null alleles in each cohort, using the population inbreeding model (PIM), a maximum likelihood approach implemented in the INEST 2.2 software (Chybicki, 2017). The inferred data of genetic diversity (NA_E_, *A*_R_, *H*_E_, *H*_O_, *F*) per locus and cohort in each population, clustered in two groups (NCP and CP), were used for statistical analyses, using an analysis of variance procedure in R (R Core Team, 2013). Differences between CP and NCP were assessed in reproductive trees by performing a bootstrap procedure for independent samples, as implemented in SPSS 22.0 for Windows (IBM, Armonk, NY, USA). The following parameters for bootstrap analysis were set: stratified sampling method, 1000 bootstrap samples, 95% confidence interval, and bias‐corrected and accelerated interval for confidence interval type.

### Fine‐scale spatial genetic structure analyses

Spatial genetic structure was characterized relying on the spatial autocorrelation between pairs of adult individuals *i* and *j* based on kinship coefficient *F_ij_* (Loiselle et al., 1995) using SPAGeDi 1.4 (Hardy and Vekemans, 2002). The spatial autocorrelation analysis was performed using eight distance classes (0–100; 101–200; 201–400; 401–800; 801–1600; 1601–3200; 3201–6400; 6401–10,000) for Cassou and Saki (NCP) and seven distance classes (0–200; 201–400; 401–800; 801–1600; 1601–3200; 3201–6400; 6401–10,000) for Vouza and Walley (CP), the lower‐density populations. The presence of structuring at each distance class was tested by randomly permuting 10,000 times the spatial positions of individuals. We estimated standard errors by using jackknifed estimators over loci. Indirect estimates of neighborhood size (*N*_b_) and the strength of spatial genetic structure (*S*_p_) were quantified by *S*_p_ = *b*_F_/(*F*_1_ − 1) and *N*_b_ = (F_1_ − 1)/*b*_F_, where *F*_1_ is the average pairwise kinship coefficient at the first distance class and *b*_F_ is the slope of the logarithm regression (Vekemans and Hardy, 2004).

### Mating system analyses

Mating system parameters were estimated using the mixed mating model as implemented in the software MLTR 3.4 (Ritland, 2002). Multilocus outcrossing rate (*t*_m_) and single‐locus outcrossing rate (*t*_s_) were calculated from progeny arrays using known maternal parents and the Newton–Raphson estimation procedure. Selfing rate (*s*) was also assessed by NMπ software (Chybicki, 2018). The standard error of mating system estimates was evaluated using 1000 bootstraps. The difference between *t*_m_ and *t*_s_ gives an indirect estimation of the presence of biparental inbreeding. The difference in means of mating system parameters between CP and NCP was assessed using a bootstrap procedure for independent samples in SPSS with the same parameters set above.

### Pollen‐mediated dispersal characteristics

We relied on different methodologies to compare patterns of pollen‐mediated gene flow between CP and NCP. Pollen dispersal parameters were first inferred through an indirect procedure using KINDIST and TWOGENER programs, as implemented in POLDISP software (Robledo‐Arnuncio et al., 2007). In each population, our analyses considered mother–offspring genotypic data (NCP: Saki 18‐408, Cassou 20‐456; CP: Walley 19‐419, Vouza 20‐464), as well as genotypes of all adult trees as potential fathers (NCP: Saki 212, Cassou 142; CP: Walley 174, Vouza 175). The KINDIST program was first used to estimate the correlated paternity rate of outcrossed progenies within each maternal sibship and between each maternal sibship pair and the average distance in meters of pollen dispersal from the mapped genotypes of mother‐offspring data. From the within‐sibship‐correlated paternity (*r*_p_, which is the probability of sharing the same father within a maternal sibship), we calculated the mean effective number of pollen donors that participate in cross‐pollination as *N*_EP_ = 1/*r*_p_. We tested whether the among‐sibship‐correlated paternity is inversely correlated with the distance between mother trees using a Mantel test procedure as implemented in the zt software (Van de Peer, 2002). For each population, the slope was negative and significant. The different pollen dispersal distribution models available in POLDISP (normal, exponential, exponential power, geometric, 2Dt) were then tested for each population. Reference threshold distances were set to 50, 200, 275, and 300 m in Saki, Cassou, Walley, and Vouza, respectively, as there was no decrease of the among‐sibship correlated paternity beyond these distances. Second, we used the TWOGENER program to estimate pollen dispersal parameters using an exponential‐power dispersal distribution as the assumed dispersal kernel because it provided a better fit to data as demonstrated by least‐square residual values. The effective male population density (*D*_EP_) was estimated with TWOGENER using as input the scale parameter of the dispersal distribution (*a*) as estimated with KINDIST (Appendix S5). The ratio *D*_EP_/*D* provides an indication of the proportion of adult trees that contributed to reproduction as pollen donors within the population.

A paternity analysis was then conducted to directly infer pollen dispersal using the CERVUS program (Marshall et al., 1998), considering the genotypes of maternal trees, seeds, and adults (from the plot and the 5‐km paths). Analyses were carried out separately for each population. The paternity of offspring was assigned based on the critical logarithm of odds (LOD) score obtained during the simulation of paternity analysis with the following parameters: 10,000 simulated mating events, all adults of the given population included as candidate fathers, individuals typed at a minimum of eight loci, the proportion of candidate fathers and typing error set at 0.5 and 0.05, respectively. The most likely candidate parent pair was assigned for parentage at 95% confidence level, considering a maximum of two mismatches among the offspring, maternal tree, and putative pollen donor trio. The effective pollen dispersal distance was estimated based on position (*x_i_, y_i_*) of maternal tree *i* and position (*x_j_, y_j_*) of putative pollen donor *j* assigned by paternity analysis. Pollen dispersal distance *δ*_p_ was calculated considering the Euclidean distance between the two spatial coordinates: $\delta_{p}\sqrt{{\left(x_{i}‐x_{j}\right)}^{2}+{\left(y_{i}‐y_{j}\right)}^{2}}$.

Characteristics of the pollen dispersal kernel were then evaluated using the neighborhood model as implemented in NMπ. This method allows estimating through a maximum likelihood approach: (1) genotyping error rates per locus; (2) selfing rate (*s*); (3) pollen immigration rates (*m*_p_), which provides an indication on the proportion of seeds pollinated by nonsampled adults (i.e., fathers outside the plot); (4) mean distance of pollen dispersal (*d*_p_); (5) shape parameter of pollen dispersal kernel (*b*_p_) (*b* < 1 for a fat‐tailed distribution, *b* = 1 for an exponential distribution, *b* = 2 for a Gaussian distribution); and (6) the effect of DBH on male reproductive success. For each population, analyses were run on two data sets: (1) all adults from the plot only (used to estimate *m*_p_); (2) all adults from the plots and the transects (used to estimate other characteristics of the pollen kernel, notably the shape parameter that is more accurately estimated using the whole data set).

Finally, we estimated the male reproductive success by studying the relationship between the number of times an individual has been identified as a male tree by paternity analysis (number of pollination events it has contributed to) and its diameter at breast height (DBH). To this aim, we used a generalized linear model (GLM) with negative binomial distribution and log‐link. Covariates were the DBH, its quadratic term and the interaction with the respective tree population. The DBH has been centered to its sample mean value (48.7).

To test differences in mean values of pollen dispersal (*r*_p_, *N*_EP_, *δ*_p_) between CP and NCP, we performed a bootstrap procedure for independent samples using SPSS as above based on pollen dispersal data inferred per maternal trees in each population from KINDIST and CERVUS.

## Results

### Genetic diversity and inbreeding

The genetic diversity and inbreeding analyses of *P. biglobosa* focused on 2468 individuals including seedlings, embryos, and adult trees across the four populations. Seedlings from Saki were not used in the analyses due to the limited sample size (Table 1; Appendix S2). The genotyping indicated 201 alleles were harbored in total in the four populations, with an average of 20 alleles per locus (Appendix S3). The multilocus mean estimates of NA_E_, *A*_R_, *H*_E_ and *H*_O_ were 6.27, 9.91, 0.81 and 0.79, respectively (Appendix S3).

Overall, the inbreeding coefficient was close to zero in all cohorts investigated (embryos, small trees and large trees) in all studied populations (between −0.052 and 0.035; Table 2). The inbreeding coefficient was significantly different from zero (*p* < 0.001) only in the embryo and mature tree cohorts from Saki, probably in relation to the presence of null alleles (Table 2). For each population, the level of inbreeding was thus similar (null) among cohorts: we did not observe an increase in inbreeding from adult to embryo cohort in CP. Three loci (PbL04, PbL09, PbL22) presented a signal of heterozygosity deficit at Saki, Walley, and Vouza (Appendix S3). This signal disappeared when controlling for the presence of null alleles (using INEST, Table 2). Overall, levels of genetic diversity were high and inbreeding coefficients were close to zero in populations and cohorts (Table 2; Appendices S3 and S4). Genetic diversity estimates tended to increase from embryos to large trees in studied populations, but this trend was supported statistically only for allelic richness (Table 2) and could be due to a sampling bias (too few mother trees sampled per population) as verified by simulation analyses (data not shown). However, mean differences in genetic diversity (NA_E_, *A*_R_, *H*_E_, *H*_O_) and inbreeding (*F*) between NCP and CP were not significant (Appendix S4).

**Table 2:** Difference of genetic diversity parameters among cohorts within *Parkia biglobosa* populations.

Populations	Cohorts	*N*	NA_E_	*A*_R_ (*k* = X)	*H*_E_	*H*_O_	*F*	*F*_(null)_
**Non‐cotton populations (NCP)**								
Saki	Embryos	408	5.44	11.68	0.78	0.76	0.027***	0 (0)
	Small trees	68	5.91	14.03	0.8	0.79	0.019	0 (0)
	Large trees	144	6.04	13.98	0.81	0.78	0.035**	0 (0)
	Difference among cohorts		NS	***	*	NS	NS	
Cassou	Embryos	456	5.47	8.7	0.79	0.79	–0.008	0 (0)
	Seedlings	18	4.55	8.2	0.74	0.78	–0.052	0 (0)
	Small trees	54	5.22	8.88	0.78	0.77	0.007	0 (0)
	Large trees	88	5.74	9.38	0.8	0.79	0.023	0 (0)
	Difference among cohorts		NS	***	NS	NS	NS	
**Cotton populations (CP)**								
Walley	Embryos	419	5.79	10.66	0.79	0.8	–0.002	0 (0)
	Small trees	37	6.28	11.73	0.8	0.77	0.037	0 (0)
	Large trees	137	6.57	12.23	0.81	0.8	0.016	0 (0)
	Difference among cohorts		NS	***	NS	NS	NS	
Vouza	Embryos	464	6.13	6.89	0.81	0.8	0.004	0 (0)
	Small trees	8	5.89	5.8	0.81	0.8	0.019	0 (0)
	Large trees	167	6.37	7.18	0.82	0.81	0.007	0 (0)
	Difference among cohorts		NS	*	NS	NS	NS	

Notes

*N*, sample size; NA_E_, effective number of alleles; *A*_R_, rarefied allelic richness based on *k* gene copies (*k* = 132, 36, 74, 16, respectively in Saki, Cassou, Walley, Vouza); *H*_E_, expected heterozygosity; *H*_O_, observed heterozygosity; *F,* inbreeding coefficient (departures from Hardy–Weinberg proportions **p < 0.01), ***p > 0.001); *F*_(null)_, inbreeding coefficient accounting for null alleles, SE estimated by jackknife in bracket. Differences of genetic diversity among cohorts tested through two‐way analysis of variance (ANOVA) procedure (*p < 0.05, **p < 0.01; ***p < 0.001), NS, nonsignificant.

### Spatial genetic structure and inference of historical gene dispersal

**Figure 2:**
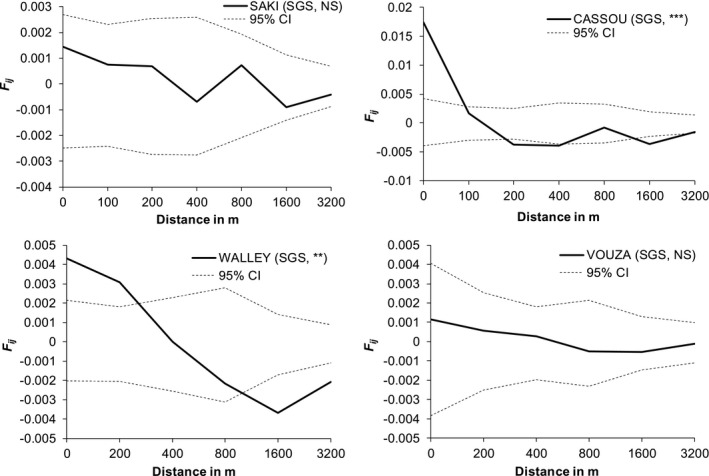
Spatial autocorrelation analysis using kinship coefficients *F_ij_* between pairs of adult individuals *i* and *j* at different geographic distance intervals in non‐cotton populations (NCP: Cassou, Saki) and in cotton populations (CP: Walley and Vouza) of *Parkia biglo*bosa. Abbreviations: Spatial genetic structure (SGS); significance of SGS: NS = not significant at the 5% level, * = significant at the 5% level, ** = significant at the 1% level, *** = significant at the 0.1% level; 95% CI = confidence interval at 95%.

A significant signal of isolation by distance was observed in Cassou (NCP) and Walley (CP) (Fig. 2). The SGS test showed that *F_ij_* was significant for the first distance class (0–100 m) in Cassou and for the two first distance classes in Walley (0–200 and 201–400 m). The pairwise kinship coefficients decreased with distance and became nonsignificantly different from zero at 100 m in Cassou (8.3 adults/ha) and 300 m in Walley (4.7 adults/ha), indicating that spatially close individuals are related at these respective distances. Overall, the strength of SGS in the four studied populations was very weak (*S*_p_ = 0.002–0.003 at Cassou and Walley and <0.001 at Saki and Vouza; Appendix S5).

### Mating system patterns

Both CP and NCP presented a predominantly outcrossed mating system when using MLTR or MNπ and a low biparental inbreeding (*t*_m_ − *t*_s_), that varied from 0.03 to 0.05 without significant differences between CP and NCP (Table 3). An exception was the tree population in Vouza, which had the lowest population density and exhibited a high biparental inbreeding estimated at 0.16 (Appendix S5).

**Table 3:** Difference of means for mating and pollen dispersal parameters between non‐cotton populations (NCP) and cotton populations (CP) of *Parkia biglobosa*.

Parameters	Non‐cotton populations		Cotton populations		*P* value and significance of mean difference
	Mean (SE)	Bca 95% CI	Mean (SE)	Bca 95% CI	
**Mating system**					
*t*_m_ (MLTR)	1.000 (0.000)	0.999–1.000	0.996 (0.003)	0.988–1.000	0.43 (NS)
*t*_s_ (MLTR)	0.946 (0.009)	0.927–0.965	0.968 (0.009)	0.949–0.986	0.12 (NS)
*t*_m_ − *t*_s_ (MLTR)	0.053 (0.009)	0.035–0.072	0.029 (0.010)	0.010–0.048	0.08 (NS)
*s* (NMπ)	0.003		0.004		
**Pollen dispersal**					
*r*_p_ (KINDIST)	0.063 (0.009)	0.048–0.080	0.068 (0.010)	0.050–0.092	0.70 (NS)
*N*_EP_ (KINDIST)	21.8 (4.3)	11.3–31.4	18.3 (4.8)	8.0–28.2	0.60 (NS)
*δ*_p_ (CERVUS) [m]	220.1 (43.2)	142.4–315.4	269.7 (65.2)	165.1–412.5	0.54 (NS)
*d*_p_ (NMπ) [m]	882		595		

Notes

*t*_m_ (MLTR), multilocus outcrossing rate using MLTR; *t*_s_ (MLTR), single‐locus outcrossing rate using MLTR; *t*_m_ − *t*_s_ (MLTR), indirect estimation of the presence of biparental inbreeding using MLTR; *s* (NMπ), selfing rate using NMπ; *r*_p_ (KINDIST), average within‐sibship correlated paternity using KINDIST; *N*_EP_ (KINDIST), average number of effective pollen donors using KINDIST; *δ*_p_ (CERVUS), average of pollen dispersal distance in meters using CERVUS; *d_p_* (NMπ), average pollen dispersal distance in meters using NMπ. Results of mean, standard error (SE) and bias‐corrected and accelerated (Bca) at 95% confidence interval (CI) after 1000 bootstrap samples. Overall, the difference of means between NCP and CP using 1000 bootstrap samples for independent samples test was not significant, NS (*P* > 0.05 for all comparisons).

### Contemporary pollen dispersal patterns

With the indirect approach implemented in the KINDIST program, the most likely pollen dispersal distribution in *P. biglobosa* was an exponential‐power distribution. The pollen dispersal kernel was fat‐tailed (*a* > 0 and *b* < 1; Appendix S5) indicating long‐distance dispersal events. The scale parameter of the dispersal distribution (*a*) and the shape parameter affecting the fatness of the tail of the dispersal distribution (*b*) were similar in all populations except Vouza. This result indicates that pollen dispersal events occur at shorter distances in this population (which is also confirmed by the NMπ analysis, see below). The estimate of pollen pool differentiation in the maternal trees was low and similar (*Φ*_ft_ = 0.03–0.04) between NCP and CP. The proportion of individuals that participate in pollination (*D*_EP_/*D*) varied from 51% (Walley) to 96% (Vouza). The average within‐sibship‐correlated paternity was also low and relatively similar between NCP and CP (*r*_p_ = 0.06 and 0.07, respectively; Table 3). Consequently, the average number of effective pollen donors per maternal tree was high and quite homogenous between NCP and CP (*N*_EP_ = 22 and 18, respectively). The average pollen dispersal distance estimated through indirect analyses based on the spatial structure of pollen pools was 81–242 m and 132–259 m in NCP and CP, respectively (Appendix S5).

**Figure 3:**
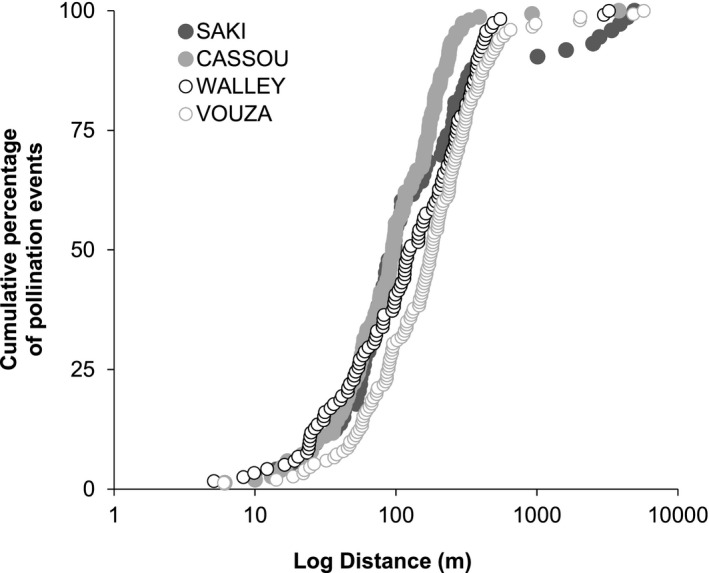
Graph of cumulative percentage of pollination events according to the logarithm of distance in the four *Parkia biglobosa* populations investigated.

The direct estimates of pollen dispersals from CERVUS indicated that all embryos were assigned to the expected maternal tree at a 95% confidence level. Given a known maternal parent, the assignment rates for a paternal parent at a 95% confidence level were 28%, 61%, 57%, and 53% at Saki, Cassou, Walley, and Vouza, respectively. The paternity analysis detected very few cases of self‐pollinated seeds in the progenies (2 in 144 seeds, 1 in 278, 3 in 224, and 1 in 248 at Saki, Cassou, Walley, and Vouza, respectively), suggesting the presence of a self‐incompatibility system in the species (Lassen et al., 2018). The confidence interval of pollen dispersal distance was 142–315 m and 165–413 m in NCP and CP, respectively (Table 3). The average pollen dispersal distance was statistically similar between NCP and CP (Table 3). The distribution of cumulative pollination events (Fig. 3) showed that pollination events tend to happen at a longer distance in the population with the lowest density (Vouza), but the comparison needs to be interpreted with caution given that results of paternity analyses are influenced by the extent of the sampling areas. The median pollen dispersal distance (excluding immigrant pollen) reached 120 m in Vouza compared to ca. 60, 70, and 80 m in Cassou, Walley, and Saki, respectively. Furthermore, in Vouza (CP) about 20% of pollination events were associated with long‐distance pollen dispersal, up to 300 m, compared to 15%, 10%, and 2% in Walley, Cassou, and Saki, respectively. Finally, the longest pollen dispersal distance was recorded at Vouza, with 5.7 km compared with 4.9, 3.8, and 3.2 km at Saki, Cassou, and Walley, respectively. These results are probably an under‐estimation of pollen efficiency in the different populations because they are based only on pollination events traced back by the paternity analyses (around half of the actual pollination events). It is probable that a great part of pollination events not captured by the analysis are not due to a lack of power of the analysis (markers used are highly polymorphic allowing a high discrimination power), but actually correspond to long‐distance dispersal events from unsampled fathers. The contribution of small trees to pollination differed slightly from one population to the other, with 28% and 21% in Saki and Cassou (NCP) against 7% and less than 1% in Walley and Vouza (CP), respectively.

NMπ analysis demonstrated that per‐locus genotyping errors were generally lower than 1%. The pollen immigration rate (*m*_p_) was the highest in Saki (the higher‐density population), with 74% of pollination events coming from pollen donors located outside of the plot (Table 4). The two CP presented similar levels of immigration, despite differences in population densities. The shape parameter of pollen dispersal kernel was lower than 0.5 in all populations, indicating fat‐tailed distributions as already demonstrated by the KINDIST analysis. The highest pollen dispersal distance was observed in Saki, followed by Walley, Cassou, and Vouza. In all populations except Vouza, the male reproductive success was correlated with the DBH of pollen donors (Table 4).

**Table 4:** Mating and pollen dispersal parameters in four *Parkia biglobosa* populations studied using NMπ.

Parameters	Non‐cotton populations		Cotton populations	
	Saki	Cassou	Walley	Vouza
Selfing rate for adult cohort (*s*)^a^	0.003 ± 0.003	0.002 ± 0.002	0.007 ± 0.004	0.002 ± 0.002
Percentage of pollen immigration rate (*m_p_*)^a^	74 ± 2	34 ± 2	46 ± 2	48 ± 2
Shape of pollen dispersal kernel (*b_p_*)^a^	0.27 ± 0.16	0.26 ± 0.07	0.25 ± 0.07	0.37 ± 0.09
Mean kernel pollen dispersal distance (*d_p_*)^b^	1428 m [537–NE]	336 m [158–NE]	900 m [370–NE]	290 m [196–564]
Effect of DBH on male fitness^a^	0.75 ± 0.09	0.43 ± 0.05	0.45 ± 0.07	–0.06 ± 1.00

^a^ Mean estimate ± SE

^b^ Mean estimate [95% CI]. NE, no estimate available.

The male reproductive success curve as a function of DBH presented a bell shape in Cassou, Saki, and Walley, whereas no clear relationship was observed in Vouza (Appendix S6). The absence of relationship in Vouza is possibly due to the low representation of small trees in this population (Appendix S1). The male reproductive success was better expressed by a quadratic relationship between DBH and the number of pollination events per tree for three populations (Cassou, Saki, and Walley). The modelling indicated in each tree population an increase in male reproductive success up to a plateau (around 60–75 cm DBH) and then a slow decrease toward the upper limit of the DBH range (Appendix S6).

## Discussion

We have characterized the genetic diversity and different aspects of *P. biglobosa* reproductive biology, focusing on four populations in different settings with respect to cotton cultivation. Cotton cultivation is known to reduce tree density in agroforestry systems and could cause a potential reduction in the activity of pollinators. Such a reduction can affect the spatial dynamics of tree species reproduction. We had expected differences in genetic diversity, mating system, and patterns of pollen‐mediated gene flow between tree populations in cotton‐ and non‐cotton‐growing areas. However, such differences were not detected in our study.

### Levels of genetic diversity

In the *P. biglobosa* populations studied, relatively high levels of genetic diversity and a low inbreeding coefficient were found. Levels of genetic diversity were equivalent among populations. Comparing genetic diversity parameters for populations belonging to different intraspecific gene pools can be problematic as each gene pool has its own evolutionary history that can differently influence the level of genetic diversity. Saki, Vouza, and Walley are from the same gene pool (CWA2), whereas Cassou corresponds to another (CWA3) (Lompo et al., 2018). However, given that the genetic diversity estimates in CWA2 and CWA3 are similar (Lompo et al., 2018) and that three of the four populations studied belong to CWA2, we consider our comparisons to be valid. Overall, our estimates of genetic diversity were high (e.g., *H*_E_ = 0.81) and similar to those found in other *P. biglobosa* populations from the central part of West Africa (Lompo et al., 2018). Genetic diversity estimates were higher than those found in other Sudanese tree species genotyped with microsatellites: *Blighia sapida* (*H*_E_ = 0.29, Ekué et al., 2011), *Khaya senegalensis* (*H*_E_ = 0.64, Sexton et al., 2015), and *Vitellaria paradoxa* (*H*_E_ = 0.42–0.62, Allal et al., 2011; *H*_E_ = 0.57–0.71, Logossa et al., 2011).

We did not observe a decrease of genetic diversity or increase in inbreeding from adult to embryo cohorts as one may expect in populations affected by a drastic decline in pollination service, as hypothesized under cotton cultivation. Such a result needs to be considered with caution, as the direct and indirect effects of cotton cultivation on genetic diversity might only be visible after multiple tree generations. A first worrying signal is the absence of regeneration in CP. The population structure of CP can be a consequence of the fragmentation of the habitat in relation to cotton cultivation, as per a recent meta‐analysis (Aguilar et al., 2019), which has demonstrated that plant progeny showed reduced germination, survival, and growth in fragmented habitats.

### SGS patterns and historical gene flow

The strength of the fine‐scale SGS (*S*_p_ < 0.003) was relatively weak and equivalent in all populations. SGS was calculated based on the spatial distribution of genetic diversity of adult trees and is indicative of a predominantly outcrossing species. It is important to note that in Walley and Saki populations, most adult trees were already in place before the start of cotton cultivation, so the effect of cotton cultivation on SGS cannot be evaluated in these populations. It would have been interesting to compare patterns of SGS in older trees (those present before cotton cultivation started) and younger trees (those established after the start of cotton cultivation), but sample sizes would be too small.

Such weak SGS was also observed in other African forest tree species characterized by long‐distance dispersal of pollen and seeds, such as *Baillonella toxisperma* (*S*_p_ = 0.003, Ndiade‐Bourobou et al., 2010; *S*_p_ = 0.0095, Duminil et al., 2016b), *Entandrophragma cylindricum* (*S*_p_ = 0.0058; Monthe et al., 2017), *Erythrophleum suaveolens* (*S*_p_ = 0.006; Duminil et al., 2016a), and *Milicia excelsa* (*S*_p_ ~ 0.0002–0.006; Bizoux et al., 2009).

### Mating system

We found that the mating system is similar in all four populations, whatever the treatment considered. These findings suggest that cotton cultivation did not affect, as expected, the capacity of *P. biglobosa* to reproduce through cross‐fertilization. We can hypothesize that the reduction of pollinator abundance would have created a reduction of allogamy in CP. But we can also imagine that such a reduction of pollinator abundance does not have any effect on selfing, if the amount of outcross‐pollen, even being reduced, is still sufficient to ensure fertilization. It is difficult to draw conclusions because different pollinators are involved in *P. biglobosa* pollination and they may be affected by pesticides quite differently. Thus, the relative contribution of each pollinator's guild may change, but the overall pollination service may not be significantly affected. An increase in selfing was also expected from a reduction in *P. biglobosa* population densities when pollinators were not able to travel over long distances. However, in our study, the mating system was similar in all populations, despite differences in tree densities. This absence of relationship between population density and mating system has also been observed for *Erythrophleum suaveolens*, a timber species from Central Africa (Duminil et al., 2016a).

Our results reveal that the species is predominantly outcrossing (*t*_m_ ~1), with little selfing (less than 1%, as detected in paternity analyses) and with insignificant inbreeding. Previous studies based on allozyme markers and a controlled pollination experiment showed that *P. biglobosa* is self‐compatible with a selfing rate of 5% (Ouedraogo, 1995; Sina, 2006). It is worth noting that Lassen et al. (2017) reported a selfing rate of 2% in a humid site with a population density of 1.23 trees/ha, similar to our lowest population density recorded in Vouza. However, a higher selfing rate (*s* ~21%) was found in a drier site with a tree population density 20 times lower than in our site with lowest tree density (0.26 trees/ha), which seems to suggest that selfing does not occur until a minimum threshold in population density is reached. On the other hand, in a humid site previously studied, selfing increased from 2 to 19% when both bats and honey bees were excluded (Lassen et al., 2017), demonstrating the role of these species in supporting cross‐pollination. In addition, our study estimated a number of pollen donors per maternal tree from 3 to 20 times higher than in the experiment by Lassen et al. (2017).

The relationship between tree density and mating system is probably very complex, due to the mix of tree species occurring in parklands and the large diversity of species involved in pollinating *P. biglobosa*. An interesting next step in this research would be to assess the relative contribution of different pollinators to the reproduction of *P. biglobosa* in the four populations studied, to better interpret our results and relate the findings with the potential influence of pesticide use in areas with cotton cultivation. The mating system of CP and NCP was found to be similar, despite differences in tree density. If important pollinators such as *Apis mellifera* were affected by pesticide use, we would have detected higher selfing rates within rather than outside the cotton cultivation areas. The similarity in mating system of CP and NCP suggests that use of pesticides does not cause a strong decline of pollination services, either because pollinating species are not sensitive to the pesticides used or because some mitigating mechanisms are in place by which a few pollinating species are able to effectively sustain pollination services. These are open questions that deserve further investigation, along the line of research conducted by Lassen et al. (2017) on pollinator behavior.

### Contemporary gene flow

We obtained a low pollen pool differentiation for all populations (*Φ*_FT_ ~ 0.03; Appendix S5), and we did not detect any significant differences between CP and NCP. This estimate of pollen pool differentiation is indicative of a high effective number of pollen donors per maternal tree (*N*_EP_ = 1/2*Φ*_FT_) according to the pollen structure model in POLDISP, which roughly corresponds to estimation using MLTR (*N*_EP_ ~18–22). We obtained a similar range of pollen dispersal distances using either indirect (KINDIST: *δ*_p_ ~82–259 m) or direct (CERVUS: *δ*_p_ ~220–270 m) analyses (Appendix S5). Results correspond roughly to the historical gene flow distance that we obtained. However, the estimates of pollen dispersal distances present differences among the four *P. biglobosa* populations studied, depending on the analytical method used (e.g., in Saki, we recorded the lowest estimate using KINDIST, and the highest estimate using CERVUS; Appendix S5). Overall, estimates by direct methods were slightly higher than those obtained through indirect methods. This discrepancy cannot really be interpreted because KINDIST does not provide confidence intervals around estimates of dispersal distance. Furthermore, results obtained using NMπ bring another contradiction. Mean pollen dispersal distances obtained by CERVUS are a priori less accurate than distances obtained by NMπ. When the sampling plot size is inferior to species pollen dispersal capacity, we expect estimates obtained by CERVUS to be inferior to estimates obtained by NMπ. Both methods are based on paternity analyses and are thus affected by the sampling scheme: if the size of the plot where exhaustive sampling is conducted is too small, the number of pollination events detected will be strongly reduced (notably long‐distance dispersal events). Given the immigration rates obtained with NMπ, there is no doubt that the size of our plots is too small to accurately characterize gene flow in *P. biglobosa*, as in three populations a confidence interval could not be estimated (Table 4). However, NMπ further explored results obtained by the paternity analysis by adjusting a pollen dispersal kernel curve to these paternity attributions. Doing so partly integrated the percentage of pollination events at long distance that had not been detected by paternity analyses, and slightly improved the estimation of mean pollen dispersal distances. Despite these limitations, the two direct methods used outperform estimated mean pollen dispersal distances by using KINDIST. Setting aside these methodological aspects, we did not find any relationship between pollen dispersal distance and cotton cultivation, despite the large differences in tree densities outlined above, between CP and NCP populations. Duminil et al. (2016a) have shown that a lower population density can be compensated by a longer distance travelled by pollinators. This relationship is not supported by our results. Nevertheless, the results show that pollen can travel long distances given the effective connectivity between maternal trees in the plot and pollen donors sampled along transect. The reproductive success of *P. biglobosa* in the low‐density populations suggests that the influence of a reduced tree density on mating is somehow buffered by other factors. One of these could be the effective pollination services provided by bats, which are capable of transporting pollen over long distances. Previous studies have demonstrated that vertebrate pollinators, such as bats and birds, can mitigate a reproductive decline in fragmented forest landscapes (Byrne et al., 2007; Aguilar et al., 2019).

Our study does not allow us to conclude whether cotton cultivation has any impact on specific pollinators, because we do not have data on the presence/absence of specific pollinators in each population studied, particularly considering that many pollinators are involved in *P. biglobosa* reproduction (Lompo et al., 2017). If insects are affected by the use of pesticides used in cotton fields (to be investigated), we cannot rule out that a compensation is occurring from the role played by bats in species pollination. It would be interesting to investigate the abundance and the respective contribution of the different cohort of pollinators involved in the pollination of *P. biglobosa* to better interpret the apparent lack of difference between CP and NCP observed in this study. The average number of effective pollen donors is relatively similar between NCP and CP, meaning that if pollinator cohorts diverged among the two treatments (e.g., with the absence of bees in CP), the consequences on population reproductive dynamics cannot be demonstrated. Moreover, male reproductive success was the highest for trees with DBH 60–75 cm in three of the four populations (no clear pattern emerged in Vouza; Appendix S6), and small trees poorly participated toward pollination. If cotton cultivation were to be expanded to additional forested areas, reducing tree density, we would recommend keeping large trees for their strong contribution to current species regeneration and small trees as future contributors. It is worth noting that all studied populations demonstrated problems of regeneration, but this problem seems much more pronounced in CP, where no seedlings were found in our samples.

## Conclusions

Our study suggests that, in the study sites investigated, the reduced density of *P. biglobosa* stands in areas characterized by cotton cultivation and the associated use of pesticides did not have a substantial effect on the levels of genetic diversity of the tree species, its mating system, and pollen dispersal and did not lead to significant inbreeding. Given that the development of cotton cultivation is relatively new compared to tree species generation time, we cannot rule out that the decline in pollination services, as estimated under cotton cultivation, is not yet observable. Moreover, we are currently lacking data on the abundance and relative contribution of the different cohorts of pollinators involved in the reproduction of *P. biglobosa*. Estimating the influence of pesticide use on pollination patterns would require a systematic inventory of the presence/absence of different pollinators involved in the reproduction of *P. biglobosa* in the different sites investigated. Hence, we suggest further investigations at a different scale on the reproductive biology of *P. biglobosa*, focusing especially on pollinators, population phenology (flowering and fruiting), landscape fragmentation, and the mechanism of self‐incompatibility.

We note again that the regeneration of two CP seems to be affected because we could not find seedlings in these populations. If the same trend is confirmed, these populations will be strongly affected in upcoming years. We should better understand the reproductive biology of this very important tree species exposed to significant land‐use changes.

Given ongoing threats to agroforestry trees in the Sudanian region of West Africa, in situ and ex situ conservation strategies need to be developed. For ex situ strategies, it should be considered that all seeds in a pod are full siblings (Lassen et al., 2014) and that one seed tree is generally pollinated by multiple pollen donors. Hence, we recommend collecting many separate pods (more than 25) through the whole crown of each tree.

## Author contributions

T.G. and B.V. designed the study, D.L. collected the samples, D.L. and H.K. performed lab analyses, and D.L. and J.D. analyzed the data and wrote the first draft of the manuscript. All authors contributed critically to the drafts and approved the final version of the manuscript for publication.

## Supporting information


**APPENDIX S1.** Diameter structure (number of individuals per DBH category) of the four *Parkia biglobosa* populations.

**APPENDIX S2.** Spatial distribution of *Parkia biglobosa* showing location of individual trees in all study sites.

**APPENDIX S3.** Genetic diversity and inbreeding coefficient in each of the four populations and per locus based on 2468 genotypes of *Parkia biglobosa*.

**APPENDIX S4.** Parameters of spatial genetic structure, mating system, and pollen dispersals in the four *Parkia biglobosa* populations investigated.

**APPENDIX S5.** Parameters of spatial genetic structure, mating system, and pollen dispersals in the four *Parkia biglobosa* populations investigated.

**APPENDIX S6.** Male reproductive success rate: relationship between the number of times an individual contributed to pollination of collected seeds (*y‐*axis), as estimated by paternity analysis, and its diameter at breast height (DBH, *x*‐axis), in each *Parkia biglobosa* population.

## Data Availability

Data available from the Dryad Digital Repository: (https://doi.org/10.5061/dryad.4xgxd2564) (Lompo et al., 2020).
