# Supplementary material for: Fine‐scale spatial genetic structure, mating, and gene dispersal patterns in Parkia biglobosa populations with different levels of habitat fragmentation
Source: Am J Bot. 2020 Jul 7;107(7):1041–53. doi: 10.1002/ajb2.1504 (PMC7496244; doi:10.1002/ajb2.1504)

**APPENDIX S1.** Diameter structure (number of individuals per DBH category) of the four *Parkia biglobosa* populations.


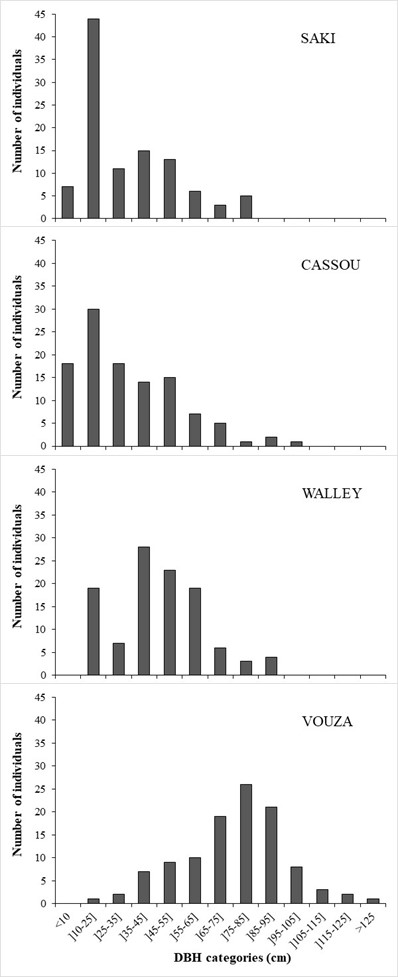

Supplement: Supplementary file 1 — APPENDIX S1. Diameter structure (number of individuals per DBH category) of the four Parkia biglobosa populations. [file AJB2-107-1041-s001.docx]
