# Supplementary material for: Fine‐scale spatial genetic structure, mating, and gene dispersal patterns in Parkia biglobosa populations with different levels of habitat fragmentation
Source: Am J Bot. 2020 Jul 7;107(7):1041–53. doi: 10.1002/ajb2.1504 (PMC7496244; doi:10.1002/ajb2.1504)

**APPENDIX S2.** Spatial distribution of *Parkia biglobosa* showing location of individual trees in all study sites.


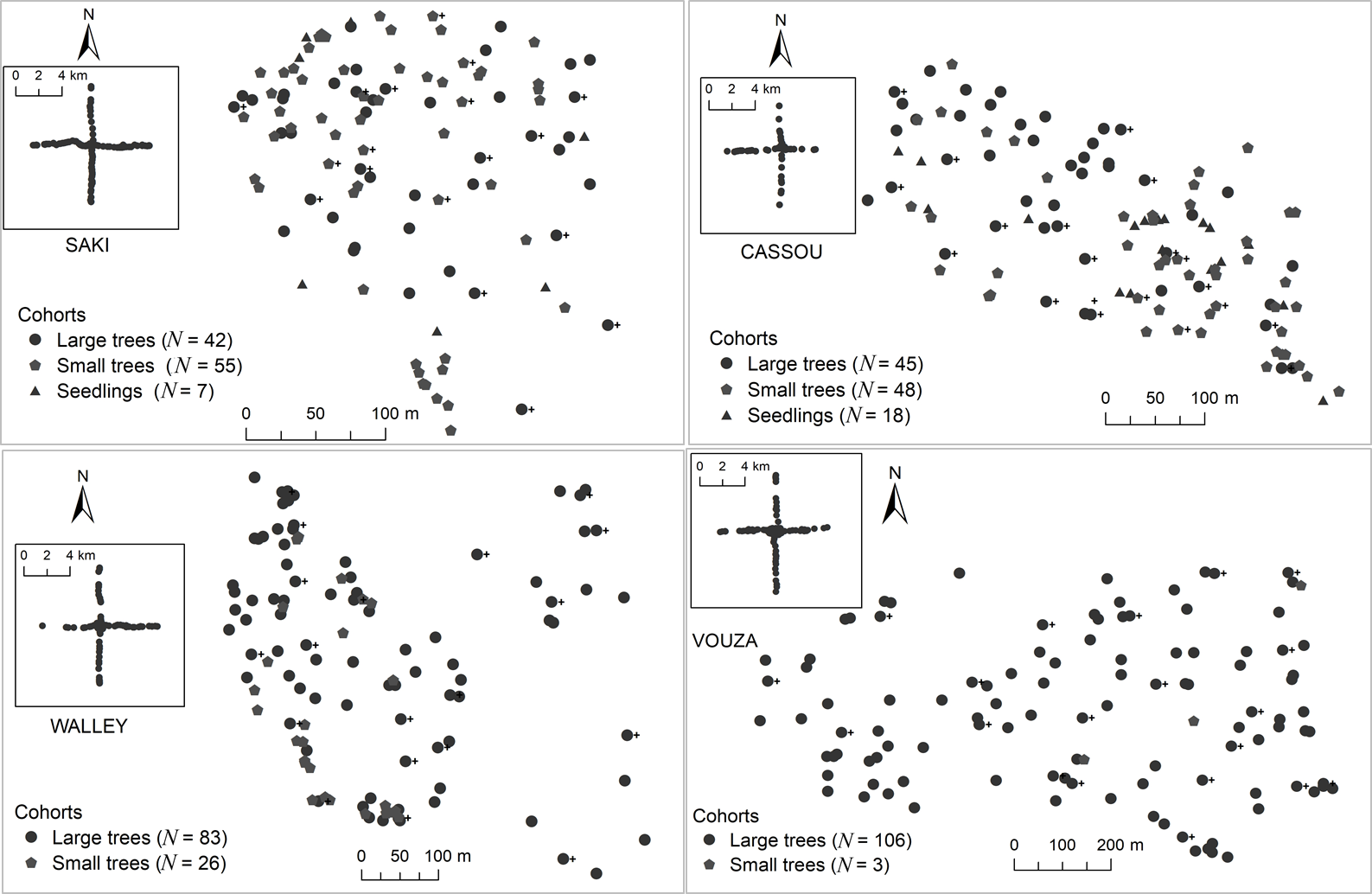

Supplement: Supplementary file 2 — APPENDIX S2. Spatial distribution of Parkia biglobosa showing location of individual trees in all study sites. [file AJB2-107-1041-s002.docx]
