# Supplementary material for: Fine‐scale spatial genetic structure, mating, and gene dispersal patterns in Parkia biglobosa populations with different levels of habitat fragmentation
Source: Am J Bot. 2020 Jul 7;107(7):1041–53. doi: 10.1002/ajb2.1504 (PMC7496244; doi:10.1002/ajb2.1504)
Supplement: Supplementary file 3 — APPENDIX S3. Genetic diversity and inbreeding coefficient in each of the four populations and per locus based on 2468 genotypes of Parkia biglobosa. [file AJB2-107-1041-s003.docx]

**APPENDIX S3.** Genetic diversity and inbreeding coefficient in each of the four populations and per locus based on 2468 genotypes of *Parkia biglobosa*.

| **Population** | **Locus** | ***N*_A_** | **NA_E_** | ***A*_R_ (*k* = 1150)** | ***H*_E_** | ***H*_O_** | ***F*_IS_** | **Frequency null alleles (SE)** |
| --- | --- | --- | --- | --- | --- | --- | --- | --- |
| **No threat of cotton production** | | | | | | | |  |
| Saki (*N* = 620) | PbL02 | 12 | 3.21 | 11.78 | 0.69 | 0.72 | -0.038 (ns) | 0.001 (0.003) |
|  | PbL03 | 22 | 9.42 | 21.71 | 0.89 | 0.91 | -0.021 (ns) | 0 (0.000) |
|  | PbL04 | 17 | 7.27 | 16.94 | 0.86 | 0.74 | 0.145 (***) | 0.078 (0.012) |
|  | PbL05 | 16 | 5.77 | 15.86 | 0.83 | 0.85 | -0.025 (ns) | 0 (0.000) |
|  | PbL09 | 21 | 7.63 | 20.87 | 0.87 | 0.8 | 0.077 (***) | 0.038 (0.009) |
|  | PbL11 | 11 | 3.21 | 11 | 0.69 | 0.69 | 0.005 (ns) | 0.001 (0.003) |
|  | PbL12 | 14 | 5.97 | 13.93 | 0.83 | 0.81 | 0.027 (ns) | 0.011 (0.008) |
|  | PbL15 | 9 | 2.52 | 8.93 | 0.6 | 0.61 | -0.01 (ns) | 0 (0.002) |
|  | PbL21 | 22 | 5.56 | 21.92 | 0.82 | 0.8 | 0.023 (ns) | 0 (0.000) |
|  | PbL22 | 24 | 6.01 | 23.78 | 0.83 | 0.75 | 0.095 (***) | 0.045 (0.009) |
|  | Multilocus | 16.8 | 5.66 | 16.67 | 0.79 | 0.77 | 0.031 (**) |  |
| **Medium threat of cotton production** | | | | | | | |  |
| Cassou (*N* = 616) | PbL02 | 11 | 4.3 | 11 | 0.77 | 0.74 | 0.033 (ns) | 0.007 (0.009) |
|  | PbL03 | 17 | 6.66 | 16.87 | 0.85 | 0.87 | -0.028 (ns) | 0 (0.000) |
|  | PbL04 | 13 | 4.09 | 13 | 0.76 | 0.76 | -0.01 (ns) | 0.001 (0.005) |
|  | PbL05 | 14 | 6.01 | 13.99 | 0.83 | 0.86 | -0.026 (ns) | 0 (0.000) |
|  | PbL09 | 22 | 8.65 | 21.66 | 0.88 | 0.87 | 0.013 (ns) | 0.002 (0.006) |
|  | PbL11 | 12 | 4.23 | 11.93 | 0.76 | 0.77 | -0.005 (ns) | 0.002 (0.004) |
|  | PbL12 | 13 | 4.71 | 12.93 | 0.79 | 0.8 | -0.016 (ns) | 0 (0.000) |
|  | PbL15 | 8 | 2.31 | 8 | 0.57 | 0.55 | 0.023 (ns) | 0 (0.001) |
|  | PbL21 | 19 | 8.71 | 18.86 | 0.89 | 0.9 | -0.016 (ns) | 0 (0.000) |
|  | PbL22 | 27 | 4.98 | 26.73 | 0.8 | 0.78 | 0.023 (ns) | 0.004 (0.006) |
|  | Multilocus | 15.6 | 5.46 | 15.5 | 0.79 | 0.79 | -0.002 (ns) |  |
| **High threat of cotton production** | | | | | | | |  |
| Walley (*N* = 593) | PbL02 | 12 | 3.95 | 12 | 0.75 | 0.72 | 0.031 (ns) | 0.010 (0.009) |
|  | PbL03 | 21 | 8.78 | 20.98 | 0.89 | 0.9 | -0.014 (ns) | 0 (0.000) |
|  | PbL04 | 17 | 7.7 | 16.98 | 0.87 | 0.85 | 0.027 (ns) | 0.014 (0.007) |
|  | PbL05 | 15 | 7.77 | 15 | 0.87 | 0.88 | -0.006 (ns) | 0.000 (0.000) |
|  | PbL09 | 22 | 8.38 | 22 | 0.88 | 0.85 | 0.03 (*) | 0.016 (0.007) |
|  | PbL11 | 12 | 4.65 | 11.94 | 0.78 | 0.76 | 0.038 (ns) | 0.014 (0.009) |
|  | PbL12 | 13 | 3.9 | 13 | 0.74 | 0.75 | -0.009 (ns) | 0.001 (0.003) |
|  | PbL15 | 8 | 2.2 | 8 | 0.55 | 0.57 | -0.047 (ns) | 0 (0.000) |
|  | PbL21 | 22 | 6.75 | 21.98 | 0.85 | 0.86 | -0.007 (ns) | 0.004 (0.005) |
|  | PbL22 | 24 | 6.8 | 24 | 0.85 | 0.82 | 0.038 (*) | 0.019 (0.008) |
|  | Multilocus | 16.6 | 6.09 | 16.59 | 0.8 | 0.8 | 0.01 (ns) |  |
| Vouza (*N* = 639) | PbL02 | 12 | 3.24 | 11.89 | 0.69 | 0.71 | -0.032 (ns) | 0 (0.000) |
|  | PbL03 | 17 | 8.9 | 16.9 | 0.89 | 0.9 | -0.008 (ns) | 0 (0.000) |
|  | PbL04 | 17 | 8.82 | 17 | 0.89 | 0.82 | 0.075 (***) | 0.028 (0.009) |
|  | PbL05 | 16 | 5.85 | 16 | 0.83 | 0.84 | -0.014 (ns) | 0 (0.000) |
|  | PbL09 | 19 | 8.8 | 18.81 | 0.89 | 0.86 | 0.025 (ns) | 0.013 (0.007) |
|  | PbL11 | 12 | 4.71 | 12 | 0.79 | 0.77 | 0.029 (ns) | 0.006 (0.008) |
|  | PbL12 | 15 | 6.12 | 14.89 | 0.84 | 0.85 | -0.016 (ns) | 0 (0.000) |
|  | PbL15 | 10 | 2.57 | 9.9 | 0.61 | 0.62 | -0.022 (ns) | 0 (0.000) |
|  | PbL21 | 24 | 6.96 | 23.68 | 0.86 | 0.86 | -0.006 (ns) | 0.002 (0.004) |
|  | PbL22 | 29 | 5.9 | 28.49 | 0.83 | 0.83 | 0.004 (ns) | 0.004 (0.006) |
|  | Multilocus | 17.1 | 6.19 | 16.96 | 0.81 | 0.81 | 0.005 (ns) |  |
| **All populations (*N* = 2468)** | **Multilocus** | **20.1** | **6.27** | **18.56** | **0.81** | **0.79** | **0.028 (*)** |  |

Abbreviations: *N*, sample size; *N*_A_, number of observed alleles; NA_E_, effective number of alleles; *A*_R_ (*k* = 36), rarefied allelic richness (expected number of alleles among 36 gene copies); *H*_O_, observed heterozygosity; *H*_E_, expected heterozygosity; *F*_IS_, inbreeding coefficient with significance test of the departures from Hardy–Weinberg equilibrium: ns, not significant at 5% level; *significant at 5% level; **significant at 1% level; ***significant at 0.1% level; SE, standard error.
