# Supplementary material for: Fine‐scale spatial genetic structure, mating, and gene dispersal patterns in Parkia biglobosa populations with different levels of habitat fragmentation
Source: Am J Bot. 2020 Jul 7;107(7):1041–53. doi: 10.1002/ajb2.1504 (PMC7496244; doi:10.1002/ajb2.1504)
Supplement: Supplementary file 4 — APPENDIX S4. Parameters of spatial genetic structure, mating system, and pollen dispersals in the four Parkia biglobosa populations investigated. [file AJB2-107-1041-s004.docx]

**APPENDIX S4.** Parameters of spatial genetic structure, mating system, and pollen dispersal in the four *Parkia biglobosa* populations investigated.

| **Genetic diversity parameters** | **Non-cotton populations** | |  | **Cotton populations** | | ***P*-value** |
| --- | --- | --- | --- | --- | --- | --- |
|  | Mean (SE) | Bca 95% CI |  | Mean (SE) | Bca 95% CI |  |
| NA | 16.24 (1.07) | 14.35–18.18 |  | 16.63 (1.12) | 14.63 to 18.74 | 0.81 |
| NA_E_ | 6.09 (0.4) | 5.31–6-87 |  | 6.61 (0.51) | 5.60 to 7.66 | 0.44 |
| *A*_R_ (*k* = 284) | 15.83 (1.0) | 13.95–17.86 |  | 16.22 (1.05) | 14.32 to 18.26 | 0.80 |
| *H*_E_ | 0.82 (0.01) | 0.80–0.85 |  | 0.83 (0.02) | 0.79 to 0.86 | 0.76 |
| *H*_O_ | 0.81 (0.02) | 0.78–0.83 |  | 0.82 (0.02) | 0.79 to 0.85 | 0.65 |
| *F* | 0.019 (0.011) | 0.000–0.040 |  | 0.013 (0.013) | −0.11 to 0.038 | 0.74 |

*Abbreviations:* *N*_A_, number of observed alleles; NA_E_, effective number of alleles; *A*_R_ (*k* = 284), rarefied allelic richness based on k gene copies; *H*_E_, expected heterozygosity; *H*_O_, observed heterozygosity; *F*, inbreeding coefficient; SE, standard error; Bca, bias-corrected and accelerated; CI, confidence interval after 1000 bootstrap samples; *p*-value for comparison of means between NCP and CP using 1000 bootstrap samples.
