# Supplementary material for: Fine‐scale spatial genetic structure, mating, and gene dispersal patterns in Parkia biglobosa populations with different levels of habitat fragmentation
Source: Am J Bot. 2020 Jul 7;107(7):1041–53. doi: 10.1002/ajb2.1504 (PMC7496244; doi:10.1002/ajb2.1504)
Supplement: Supplementary file 5 — APPENDIX S5. Parameters of spatial genetic structure, mating system, and pollen dispersals in the four Parkia biglobosa populations investigated. [file AJB2-107-1041-s005.docx]

**APPENDIX S5.** Parameters of spatial genetic structure, mating system, and pollen dispersals in the four *Parkia biglobosa* populations investigated.

| **Parameters** | **Non-cotton populations** | |  | **Cotton populations** | |
| --- | --- | --- | --- | --- | --- |
|  | **Saki** | **Cassou** |  | **Walley** | **Vouza** |
| **Spatial genetic structure** | | | | | |
| *F_1_* | 0.03 | 0.012 |  | 0.02 | 0.007 |
| *S*_p_ | 0.001 | 0.003 |  | 0.002 | 0.0002 |
| **Mating system** | | | | | |
| *t*_m_ (MLTR) | 0.995 (0.067) | 0.998 (0.096) |  | 0.993 (0.067) | 1.200 (0.088) |
| *t*_s_ (MLTR) | 0.972 (0.008) | 1.002 (0.011) |  | 0.995 (0.020) | 1.039 (0.030) |
| *t*_m_ − *t*_s_ (MLTR) | 0.023 (0.065) | −0.005 (0.094) |  | −0.002 (0.052) | 0.161 (0.077) |
| **Pollen dispersal** | | | | | |
| *a* (KINDIST) | 0.029 | 0.029 |  | 0.025 | 29.125 |
| *b* (KINDIST) | 0.272 | 0.249 |  | 0.245 | 0.718 |
| Global *Φ*_ft_ (POLDISP) | 0.027 | 0.036 |  | 0.038 | 0.032 |
| *D*_EP_ (POLDISP) | 38.01 | 4.36 |  | 2.4 | 1.73 |
| *D*_EP_/*D* (%) | — | 53 |  | 51 | 96 |
| *r*_p_ (KINDIST) | 0.052 (0.012) | 0.074 (0.014) |  | 0.079 (0.019) | 0.059 (0.009) |
| *N*_EP (_KINDIST) | 23.8 (8.6) | 20.01 (2.9) |  | 17.81 (6.2) | 18.75 (7.4) |
| *δ*_p_ (KINDIST) | 81.8 | 242.6 |  | 258.7 | 132.6 |
| *δ*_p_ (CERVUS) | 526.5 (182.5) | 116.9 (28.5) |  | 221.7 (67.3) | 304.1 (94.0) |

*Abbreviations:* *F*_1_, average pairwise kinship coefficient at the first distance class; *S*_p_, strength of spatial genetic structure; *a* (KINDIST): scale parameter of the dispersal distribution using KINDIST; *b* (KINDIST): shape parameter affecting the fatness of the tail of the dispersal distribution using KINDIST; Global *Φ*_ft_ (POLDISP): pollen pool differentiation in POLDISP; *D*_EP_ (POLDISP): estimated effective density of pollen donors per hectare using KINDIST and TWOGENER; *D*_EP_/*D* (where *D* is the density of adults per hectare): proportion of individuals that participate in the pollination, *r*_p_ (KINDIST): average within-sibship correlated paternity using KINDIST; NEP (KINDIST): average number of effective pollen donors using KINDIST; *δ*_p_ (KINDIST): average of pollen dispersal distance in meters using KINDIST; *δ*_p_ (CERVUS): average of pollen dispersal distance in meters using CERVUS. The standard error of parameters is in parentheses.
