# Supplementary material for: Fine‐scale spatial genetic structure, mating, and gene dispersal patterns in Parkia biglobosa populations with different levels of habitat fragmentation
Source: Am J Bot. 2020 Jul 7;107(7):1041–53. doi: 10.1002/ajb2.1504 (PMC7496244; doi:10.1002/ajb2.1504)

**APPENDIX S6.** Male reproductive success rate: relationship between the number of times an individual contributed to pollination of collected seeds (*y*-axis), as estimated by paternity analysis, and its diameter at breast height (DBH, *x*-axis), in each *Parkia biglobosa* population. The continuous line is the GLM estimated mean reproductive success; the dashed lines are the 95% confidence interval for the mean.


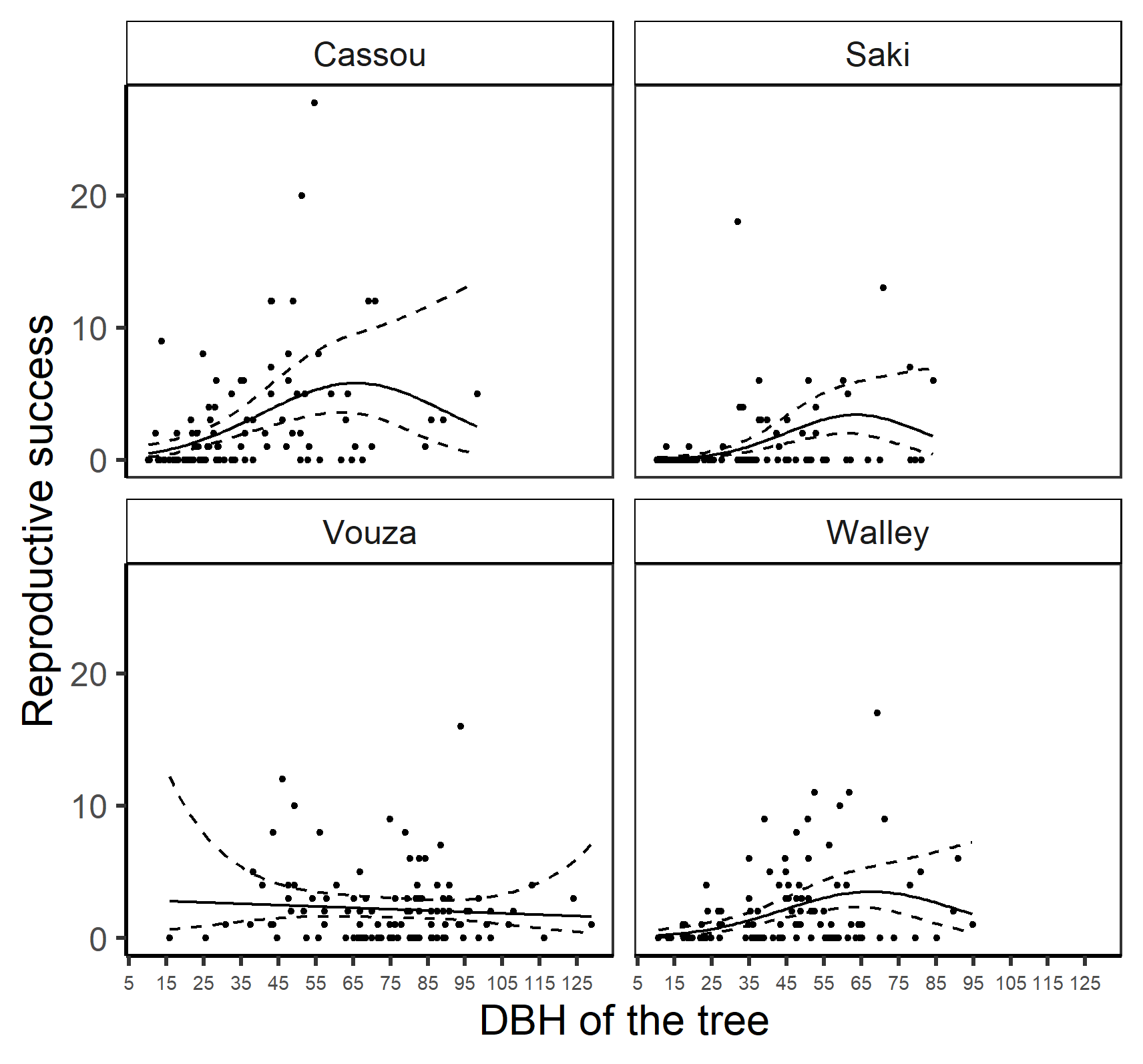

Supplement: Supplementary file 6 — APPENDIX S6. Male reproductive success rate: relationship between the number of times an individual contributed to pollination of collected seeds (y‐axis), as estimated by paternity analysis, and its diameter at breast height (DBH, x‐axis), in each Parkia biglobosa population. [file AJB2-107-1041-s006.docx]
